# Effects of acute and 14-day coenzyme Q10 supplementation on exercise performance in both trained and untrained individuals

**DOI:** 10.1186/1550-2783-5-8

**Published:** 2008-03-04

**Authors:** Matthew Cooke, Mike Iosia, Thomas Buford, Brian Shelmadine, Geoffrey Hudson, Chad Kerksick, Christopher Rasmussen, Mike Greenwood, Brian Leutholtz, Darryn Willoughby, Richard Kreider

**Affiliations:** 1 Exercise & Sport Nutrition Lab; Center for Exercise, Nutrition and Preventive Health; Department of Health, Human Performance & Recreation; Baylor University; Waco, TX, USA; 2 Department of Health, Exercise Science, and Secondary Education; Lee University; Cleveland, TN, USA; 3 Department of Health and Exercise Science; University of Oklahoma; Norman, OK, USA

## Abstract

**Background:**

To determine whether acute (single dose) and/or chronic (14-days) supplementation of CoQ10 will improve anaerobic and/or aerobic exercise performance by increasing plasma and muscle CoQ10 concentrations within trained and untrained individuals.

**Methods:**

Twenty-two aerobically trained and nineteen untrained male and female subjects (26.1 ± 7.6 yrs, 172 ± 8.7 cm, 73.5 ± 17 kg, and 21.2 ± 7.0%) were randomized to ingest in a double-blind manner either 100 mg of a dextrose placebo (CON) or a fast-melt CoQ10 supplement (CoQ10) twice a day for 14-days. On the first day of supplementation, subjects donated fasting blood samples and a muscle biopsy. Subjects were then given 200 mg of the placebo or the CoQ10 supplement. Sixty minutes following supplement ingestion, subjects completed an isokinetic knee extension endurance test, a 30-second wingate anaerobic capacity test, and a maximal cardiopulmonary graded exercise test interspersed with 30-minutes of recovery. Additional blood samples were taken immediately following each exercise test and a second muscle biopsy sample was taken following the final exercise test. Subjects consumed twice daily (morning and night), 100 mg of either supplement for a period of 14-days, and then returned to the lab to complete the same battery of tests. Data was analyzed using repeated measures ANOVA with an alpha of 0.05.

**Results:**

Plasma CoQ10 levels were significantly increased following 2 weeks of CoQ10 supplementation (p < 0.001); while a trend for higher muscle CoQ10 levels was observed after acute CoQ10 ingestion (p = 0.098). A trend for lower serum superoxide dismutase (SOD) was observed following acute supplementation with CoQ10 (p = 0.06), whereas serum malondialdehyde (MDA) tended to be significantly higher (p < 0.05). Following acute ingestion of CoQ10, plasma CoQ10 levels were significantly correlated to muscle CoQ10 levels; maximal oxygen consumption; and treadmill time to exhaustion. A trend for increased time to exhaustion was observed following 2 weeks of CoQ10 supplementation (p = 0.06).

**Conclusion:**

Acute supplementation with CoQ10 resulted in higher muscle CoQ10 concentration, lower serum SOD oxidative stress, and higher MDA levels during and following exercise. Chronic CoQ10 supplementation increased plasma CoQ10 concentrations and tended to increase time to exhaustion. Results indicate that acute and chronic supplementation of CoQ10 may affect acute and/or chronic responses to various types of exercise.

## Background

In recent years, Coenzyme Q10 (CoQ10) has gained considerable attention as a dietary supplement capable of influencing cellular bioenergetics and counteracting some of the damage caused by free radicals [1-4]. CoQ10 is a vitamin like, fat-soluble substance existing in all cells [5]. It is intimately involved in several important roles in the body including the transferring of electrons within the mitochondrial oxidative respiratory chain and hence, ATP production; acting as an essential antioxidant and supporting the regeneration of other antioxidants; influencing the stability, fluidity and permeability of membranes; and, stimulating cell growth and inhibiting cell death [5-7].

In clinical populations, CoQ10 has been used as a supplementary treatment for chronic diseases such as Chronic Heart Failure (CHF), muscular dystrophies, Parkinson's disease, cancer, and diabetes [8,9]. In CHF patients, a disease characterized by lower than normal CoQ10 levels, CoQ10 supplementation has shown to improve stroke volume, ejection fraction and exercise capacity in several double-blind, placebo-controlled studies [4]. In athletes, CoQ10 deficiency may also be experienced as metabolic stress and free radical formation is elevated during times of intense training [7,10,11]. Thus, given our current understanding into the functions of CoQ10 within the body, it has been hypothesized that dietary supplementation with CoQ10 may not only be beneficial to clinical patients, but also healthy active individuals who may potentially experience CoQ10 deficiency [3].

While this theory is plausible, results of studies that have evaluated the potential ergogenic value of CoQ10 in athletes have reported mixed results [4]. For example, several studies have found that CoQ10 supplementation (60–100 mg/day for 4–8 weeks) improves aerobic power, anaerobic threshold, exercise performance, and/or recovery after exercise in trained athletes and untrained individuals [12-14]. Conversely, other studies using similar dosages (60–150 mg/day for 3–8 weeks) have found no ergogenic benefit on maximal or submaximal exercise capacity in untrained and trained individuals [15-19].

One possible explanation for these inconsistent findings is that the absorption of CoQ10 into the mitochondrial membrane or non-deficit tissues is rather inefficient. In this regard, CoQ10 is a relatively large, hydrophobic molecule [20]. Therefore, absorption of CoQ10 into tissues is often slow and limited. Formulations that could maximize CoQ10 absorption would not only improve its uptake into the plasma, but potentially improve its absorption into the skeletal muscle. Recently, the bioavailability and absorptive properties of several CoQ10 preparations were examined (i.e., fast-melt tablet, effervescent tablet, soft gelatin liquid capsule, and a hard shell powdered capsule) [21]. Results indicated that the fast-melt tablet and effervescent formulation provided a more rapid delivery of CoQ10 to the blood, while exhibiting similar pharmacokinetic properties compared with the soft gel and hard capsule forms of CoQ10 [21]. These findings suggest that provision of CoQ10 in a fast-melt or effervescent form may facilitate CoQ10 delivery and uptake to the muscle. Theoretically, this may enhance bioavailability of CoQ10 and promote a greater metabolic and/or ergogenic impact. However, the effects of acute and chronic ingestion of a fast-melt form of CoQ10 on muscle CoQ10 concentrations and exercise performance is unknown. Therefore, the purpose of this study was to determine whether acute (single-dose) and chronic (14-days) supplementation with a fast-melt form of CoQ10 will affect muscle CoQ10 concentrations, improve anaerobic and aerobic performance, and/or affect markers of oxidative stress in trained and untrained individuals.

## Methods

### Subjects

Twenty-two aerobically trained and nineteen untrained male and female subjects (26.1 ± 7.6 yrs, 172 ± 8.7 cm, 73.5 ± 17 kg, and 21.2 ± 7.0%) participated in this study. Aerobically trained individuals had been training a minimum of two years for an average of 8 ± 2 hours/week, 9 ± 3 workouts/week, and averaged 68 ± 36 kilometers/week of aerobic exercise (i.e., running, cycling and/or swimming). Untrained individuals did not participate in any regular form of aerobic exercise for at least the past year.

All subjects signed informed consent documents and the study was approved by the Baylor University Institutional Review Board prior to any data collection. Subjects were not allowed to participate in this study if they reported any of the following: 1) current or past history of anabolic steroid use; 2) any metabolic disorders or taking any thyroid, hyperlipidmeic, hypoglycemic, anti-hypertensive, or androgenic medications; 3) ingested any ergogenic levels of creatine, HMB, thermogenics, ribose, pro-hormones (i.e., DHEA, androstendione, etc.) or other purported anabolic or ergogenic nutritional supplements within 2 months prior to beginning the study and to not take any additional nutritional supplement or contraindicated prescription medication during the protocol.

### Experimental design

**Figure 1:**
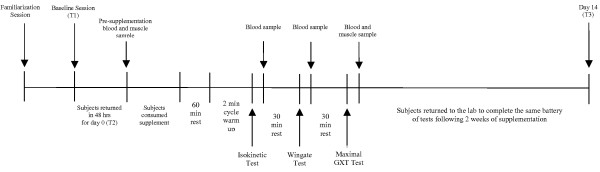
Schematic diagram of research design.

Figure 1 shows the experimental design used in this study. The study was conducted in a randomized, double-blinded, and placebo-controlled manner. All subjects eligible to participate in the study completed a familiarization session where they were provided information (both verbal and written) regarding the study design, testing and supplementation protocols. Informed consent, medical documents and training history questionnaires were also completed at this time. Subjects were then required to perform an isokinetic leg extension test and 30-second anaerobic Wingate test to become familiar with the protocol and equipment. The procedures for all exercise tests are described in detail below. Following the practice trials, subjects were scheduled to return 48 hours later for baseline testing. Subjects were asked to not change their dietary habits in any way throughout the study. This was monitored by having each subject document dietary intake for 4 days (3 weekdays and 1 weekend day) before each testing session. In addition, each subject was instructed to fast for 12 hours and not to perform any physical activity for the 48 hours preceding each testing session.

At baseline testing (T1), subjects reported to the lab in the morning to complete questionnaires regarding health status. Subjects then performed three exercise tests in the following order: 1.) isokinetic knee extension muscle endurance test; 2.) 30-second Wingate anaerobic capacity test; and, 3.) a maximal cardiopulmonary graded exercise test. Thirty minute rest periods were allocated between each exercise test. Following baseline measurements, subjects were matched based on age and aerobic capacity and randomly separated into two groups: 1.) a dextrose placebo group; or 2.) a fast-melt CoQ10 group [Fast-Melt Coenzyme Q10, Pharma Base, S.A. (Switzerland)]. Subjects were instructed to return to the lab in 48 hours for testing and ingestion of the first supplement.

On returning to the lab (Day 0, T2), subjects completed the questionnaires. Prior to ingestion of the first supplement dose, body mass, total body water, and body composition measurements were obtained. Subjects then donated approximately 20 ml of fasting blood and approximately 15–30 mg of muscle using standard procedures described below. Subjects consumed either the placebo or CoQ10 supplement. At 60 minutes following ingestion of the supplement, subjects performed the three exercise tests as mentioned above with 30 minutes of recovery between each exercise test. Additional blood samples were taken immediately following each exercise test. Subjects returned to the lab following 2 weeks of supplementation to complete the same battery of assessments. No supplement was ingested on day 14 (T3) prior to performing the exercise tests.

### Exercise protocol

#### Muscle endurance assessment

At baseline, day 0 and 14, subjects performed a 50-repetition Isokinetic leg extension test. Prior to testing, subjects warmed up on a stationary bicycle for 2–3 min at a 50–60 rpm. Subjects then performed five warm-up repetitions on the Biodex (Biodex Medical Systems, Shirley, NY, USA) isokinetic leg extension machine. After these warm-up repetitions, subjects performed unilateral leg extensions (right leg) for one set of 50 repetitions [22] at 180 degrees/s. Test-test reliability of this test has been shown to be 0.78 to 0.82 [23].

#### Anaerobic capacity assessment

At baseline, day 0, and day 14, subjects performed a 30-second Wingate anaerobic capacity test 30 minutes following completion of the muscle endurance test. Subjects warmed up for 2 min at 50–60 rpm on a stationary bicycle ergometer before performing the Wingate test. This warm-up was continued into the start of the sprinting portion of the Wingate test, which allows for a flying start. The Wingate anaerobic capacity test was performed on the LODE cycle ergometer (Amsterdam, Netherlands) with a resistance of 0.7 Nm/kg. Test-to-test variability in performing repeated Wingate tests in our laboratory yielded correlation coefficients of *r = *0.98 ± 15% for mean power.

#### Cardiopulmonary assessment

At baseline, day 0, and 14, subjects performed a volitional maximal cardiopulmonary exercise test according to the Bruce protocol 30 minutes following the anaerobic capacity test [24]. Participants were instructed to perform the test for as long as possible to ensure a true maximal attempt. Standard ACSM test termination criteria were monitored and followed throughout each test [25]. Metabolic gases were obtained with the Parvo Medics 2400 TrueMax metabolic measurement system (Sandy, UT, USA) on a Trackmaster TMX425C treadmill (Newton, KS, USA). The mean coefficient of variation (assessing maximum oxygen consumption) for this protocol was 6.5% (range, 2–14%) [24]. Ventilatory threshold (VANT) was expressed as a percentage maximal oxygen consumption (mL·kg^-1^·min^-1^). To determine VANT, data from the graded exercise test for minute ventilation (VE), ventilation equivalent of VO_2 _(VE/VO_2_), and ventilation equivalent of VCO_2 _(VE/VO_2_) were plotted against exercise time (i.e. workload) and analyzed manually by an experienced exercise physiologist in a blinded manner.

### Supplementation protocol

Subjects were assigned in a double-blind and randomized manner to ingest a dextrose placebo (CON, *n *= 20) or fast-melt CoQ10 formulation (CoQ10, *n *= 21). Subjects were matched into one of two groups according to age and aerobic capacity. Subjects were required to initially ingest 2 capsules (100 mg) prior to exercise performance testing on day 0. Following testing on day 0, subjects continued consuming 100 mg twice daily (morning and night) for 14 days. Supplements were prepared in tablet form and packaged in generic bottles for double blind administration by Pharma Base, S.A. (Switzerland). Supplementation compliance was monitored by having the subjects return empty bottles of the supplement at the end of 14 days of supplementation.

### Procedures

#### Dietary inventories

Prior to each testing session, subjects were instructed to record all food and fluid intake over a 4-day period, which was reflective of their normal dietary intake and to include one weekend day. Dietary inventories were then reviewed by a registered dietician and analyzed for average energy and macronutrient intake using the ESHA Food Processor (Version 8.6) Nutritional Analysis software (Salem, OR, USA).

#### Anthropometrics

At the beginning of every testing session, subjects had their height and body mass measured according to standard procedures using a Healthometer (Bridgeview, IL, USA) self-calibrating digital scale with an accuracy of ± 0.02 kg.

#### Body composition

Total body water was estimated using a Xitron 4200 Bioelectrical Impedance Analyzer (San Diego, CA). Whole-body composition measurements (excluding cranium) were determined with a Hologic Discovery W Dual-Energy X-ray Absorptiometer (DEXA; Hologic, Bedford, MA, USA) by using procedures previously described [26,27]. Test-retest reliability studies performed on male athletes with this DEXA machine revealed mean deviation for total bone mineral content and total fat free/soft tissue mass of 0.31% with a mean intraclass correlation of 0.985.

#### Blood collection

Subjects were required to fast for 12 hrs prior to donating approximately four teaspoons (20 milliliters) of venous blood from an antecubital vein using standard phlebotomy procedures. Blood analyzed for CoQ10 was placed in a test tube containing heparin as the anticoagulant and immediately centrifuged at 3000 g using a standard bench top centrifuge (Cole Palmer, Vernon Hills, IL, Model # 17250-10) for 15 minutes at room temperature. Plasma was separated and stored at -80°C in polypropylene tubes for later analysis. Blood analyzed for makers of oxidative stress and clinical chemistry panels were placed into two serum separation tubes and immediately centrifuged at 1100 g for 15 min. Serum was separated and stored at -80°C in polypropylene tubes for later analysis. Finally, blood was collected in a single lavender top tube containing K_2 _EDTA and refrigerated for approx 4–6 h for subsequent complete blood count with platelet differentials analysis (hemoglobin, hematocrit, red blood cell counts, MCV, MCH, MCHC, RDW, white blood cell counts, neutrophils, lymphocytes, monocytes, eosinophils, baosphils) using an Abbott Cell Dyn 3500 (Abbott Laboratories, Abbott Park, IL) automated hematology analyzer. Whole blood was only analyzed pre- and post-exercise.

#### Clinical chemistry analysis

Serum samples were analyzed using a Dade Behring Dimension RXL (Deerfield, IL, USA) automated clinical chemistry analyzer that was calibrated and optimized according to manufacturer guidelines. This analyzer has been known to be highly valid and reliable in previously published reports [28]. Each serum sample was assayed for a standard complete metabolic panel [(glucose, aspartate aminotransferase (AST), alanine aminotransferase (ALT), alkaline phosphatase (ALP), albumin, globulin, sodium, chloride, calcium, carbon dioxide, total bilirubin)], lipid panel [(triglycerides, total cholesterol, high-density lipoprotein (HDL), low-density lipoprotein (LDL), total cholesterol:HDL)] and clinical markers of protein and fatty acid metabolism [(uric acid, creatinine, blood urea nitrogen (BUN), BUN:creatinine ratio, total protein, creatine kinase (CK), ketones (betahydroxybutyrate), and lactate dehydrogenase (LDH)]. Test to test reliability (within and between) of performing these assays ranged from 2 to 6% for individual assays with an average C_*V *_of ± 3%. Samples were run in duplicate to verify results if the observed values were outside control values and/or clinical norms according to standard procedures.

#### Markers of oxidative stress

Serum samples were assayed using standard commercially available spectrophotometric assay kits (Caymen Chemical Company, Michigan, USA) for Thiobarbituric Acid Reactive Substance (TBARS, Malondialdehyde-MDA) and Superoxide Dismutase (SOD). Enzyme immunoassay (EIA) was used to measure serum 8-Isoprostane. Serum concentrations were determined using a Wallac-Victor IV (Perkin-Elmer Life Sciences, Boston, MA) micro plate reader at an optical density of 450 nm against a known standard curve using standard procedures. Test to test reliability (within and between) of performing these assays ranged from 3 to 6% for individual assays with an average C_*V *_of ± 4%.

#### Muscle sample collection

Fine Needle Aspiration (FNA) muscle biopsies were taken on days 0 and 14 prior to, and following performance tests in order to examine total CoQ10 content in the muscle according to standard procedures [29]. Participants were instructed to refrain from exercise 48 h prior to each muscle biopsy. Muscle was extracted from the lateral portion of the *vastus lateralis *midway between the patella and iliac crest of the dominant leg using a TRU-CORE^® ^1 Automatic Reusable Biopsy Instrument (Angiotech, Medical Device Technologies, INC., Gainsville, Florida, USA). Briefly, 0.5 ml of 1.0% Lidocaine HCl was injected subcutaneously prior to making a small pilot hole with a 23 gauge sterile needle. The biopsy needle was placed into the biopsy device and the inserted into the previously made pilot hole (a depth of about 5–10 mm). A muscle sample was obtained by the activation of a trigger button, which unloaded the spring and activated the needle to collect a muscle piece. The biopsy needle was removed from the pilot hole and the muscle specimen was removed from the biopsy needle using a sterile scalpel and immediately frozen in liquid nitrogen and stored at -80°C for further analyses. This whole biopsy procedure was repeated two to three times in order to obtain sufficient muscle tissue (15–30 mg).

#### CoQ10 determination

Frozen plasma and muscle samples were sent to Craft Technologies (Wilson, NC, USA) for assay of plasma and muscle CoQ10 concentrations using methods described by Tang et al., [29] and Rousseau and Varin [30]. Total CoQ10 was determined by summing the oxidized and reduced forms.

### Statistical analysis

Data are presented in all tables and throughout the text as mean (± SD). Plasma and muscle CoQ10 levels and markers of oxidative stress were analyzed using 2 × 2 × 2 × 4 (group × training status × gender × exercise [Baseline, Isokinetic, Wingate, Cardiopulmonary]) repeated measures ANOVA to assess the changes in plasma and muscle CoQ10 levels and markers of oxidative stress following acute (T2) and chronic (T3) supplementation. Delta scores (post minus pre values) were calculated for each exercise test (Isokinetic, Wingate, Cardiopulmonary) and analyzed using repeated measures ANOVA. All remaining data were analyzed using 2 × 2 × 2 × 3 (group × training status × gender × day [baseline, day 0 and 14]) repeated measures ANOVA. LSD pairwise comparisons were used to analyze any significant group × time interaction effects. Pearson product correlations were used to determine any relationship between criterion variables and an alpha level of 0.05 was adopted throughout to prevent any Type I statistical errors.

## Results

### Subject characteristics

There were no baseline differences in the age, body weight or height between the two groups (see Table 1).

**Table 1:** Subject Baseline Characteristics

**Variable**	**CoQ10**	**Placebo**
Age (yr)	25.76 ± 7.67	25.61 ± 8.07
Body Weight (kg)	76.02 ± 18.71	71.94 ± 18.12
Height (cm)	173.48 ± 7.42	168.18 ± 26.48

Data are means and ± standard deviations of mean

### Side effects

No adverse events were reported concerning the supplementation or study protocol. No reports of medical problems or side effects were indicated in post-study questionnaires administered in a blinded manner.

### Clinical safety markers

Complete blood counts with platelet differentials were run on all whole blood samples and a standard clinical safety panels was completed on all serum samples at baseline, day 0 and day 14. While some hematological variables did report main effects over time, no significant group × time interaction effects were observed and none of these changes occurred outside of the clinically accepted normative values for these variables [31]. Therefore, these data are not reported. No significant main or group × time interaction effects (p < 0.05) were found for kidney/liver enzymes [e.g., GGT (p = 0.51), AST (p = 0.14), ALT (p = 0.43), ALP (p = 0.27)] or markers of protein breakdown [e.g., uric acid (p = 0.38), billirubin (p = 0.82), creatinine (p = 0.73), BUN:creatinine ratio (p = 0.80), total protein (p = 0.30), CK (p = 0.87)].

### Lipid panels

Table 2 presents fasting lipid profile data observed throughout the study. No significant differences were reported for total (HDL and LDL) cholesterol and cholesterol:HDL ratio when all groups were included in the analysis (see Table 2). However, a significant group × time interaction was found for triglyceride levels (p < 0.05), with subsequent post-hoc analysis revealing a significant decrease in serum triglyceride levels in the CoQ10 compared to CON over the 2 week supplementation period (p < 0.05). It should be noted that triglyceride levels were below the normal range in both groups and such a decrease observed in the supplement group could primarily be due to normal physiological variation.

**Table 2:** Lipid Panels for the CoQ10 and Placebo (CON) groups.

**Variable**	**Time**	**CoQ10**	**Placebo**	**Significance**	
Total Cholesterol (mg·dl^-1^)	Day 0	165 ± 32	172 ± 31	Group	0.126
	Day 14	167 ± 25	182 ± 30	Time	0.151
				G × T	0.621
					
Triglycerides (mg·dl^-1^)	Day 0	95 ± 42	78 ± 26	Group	0.552
	Day 14	85 ± 43†	91 ± 36†	Time	0.985
				G × T	0.019
					
HDL Cholesterol (mg·dl^-1^)	Day 0	53 ± 12	58 ± 15	Group	0.443
	Day 14	54 ± 10	59 ± 15	Time	0.112
				G × T	0.296
					
LDL Cholesterol (mg·dl^-1^)	Day 0	97 ± 28	99 ± 22	Group	0.827
	Day 14	97 ± 24	104 ± 24	Time	0.004
				G × T	0.121
					
Chol:HDL	Day 0	3.19 ± 0.81	3.07 ± 0.60	Group	0.431
	Day 14	3.17 ± 0.74	3.19 ± 0.64	Time	0.183
				G × T	0.201

Data are means and ± standard deviations of mean.

† represents p < 0.05 difference between groups

### Nutritional intake and compliance

Nutritional intake was monitored over a 4-day period prior to each testing session and analyzed for energy intake, total carbohydrates, total protein and total fat (see Table 3). A significant group × time interaction was observed for total calories and protein (p < 0.05). No other statistically significant interactions were observed across groups.

**Table 3:** Nutritional Intake Analyses for the CoQ10 and Placebo (CON) groups.

**Variable**	**Time**	**CoQ10**	**Placebo**	**Significance**	
Energy Intake (kcal·kg·d^-1^)	Baseline	2591 ± 613	2103 ± 598†	Group	0.210
	Day 0	2668 ± 849	1979 ± 677†	Time	0.461
	Day 14	2514 ± 474	2227 ± 608	G × T	0.023
					
Carbohydrate Intake (g·kg·^-1^·d^-1^)	Baseline	315 ± 85	267 ± 82	Group	0.420
	Day 0	319 ± 116	247 ± 109	Time	0.410
	Day 14	318 ± 66	287 ± 72	G × T	0.132
					
Protein Intake (g·kg·^-1^·d^-1^)	Baseline	114 ± 44	99 ± 32	Group	0.112
	Day 0	124 ± 58	94 ± 34†	Time	0.243
	Day 14	104 ± 35	102 ± 12	G × T	0.012
					
Fat Intake (g·kg·^-1^d·^-1^)	Baseline	97 ± 40	70 ± 23	Group	0.120
	Day 0	104 ± 47	71 ± 28	Time	0.071
	Day 14	90 ± 36	69 ± 23	G × T	0.081

Data are means and ± standard deviations of mean.

† represents p < 0.05 difference between groups

### Body composition

Body composition was assessed at baseline, day 0 and 14 to determine changes in bone, fat and lean changes over the course of the investigation (see Table 4). No significant differences were observed between groups in changes in total body water, bone mass, lean body mass, or body fat percentage over the course of the investigation.

**Table 4:** Body Composition Measurements for the CoQ10 and Placebo (CON) groups.

**Variable**	**Time**	**CoQ10**	**Placebo**	**Significance**	
BMC	Day 0	2805 ± 694	2584 ± 487	Group	0.352
(g)	Day 14	2788 ± 706	2596 ± 452	Time	0.485
				G × T	0.219
					
Fat Mass (kg)	Day 0	15385 ± 7686	15128 ± 6307	Group	0.443
	Day 14	15252 ± 7740	15284 ± 6342	Time	0.312
				G × T	0.196
					
Lean Mass (kg)	Day 0	55274 ± 13638	51221 ± 11336	Group	0.627
	Day 14	55260 ± 13794	51311 ± 11372	Time	0.214
				G × T	0.121
					
Body Fat (%)	Day 0	21 ± 7.2	22 ± 7.0	Group	0.731
	Day 14	21 ± 7.0	22 ± 6.9	Time	0.183
				G × T	0.201

Data are means and ± standard deviations of mean

### Muscle endurance assessment

A 50 repetition isokinetic leg extension test was performed at baseline, day 0, and day 14 to assess indices of muscle endurance performance (see Table 5). No statistically significant interactions were observed among groups in indices of muscle endurance (peak torque extension [EXT], peak torque flexion [FLX], total work EXT, total work FLX, work fatigue EXT, work fatigue FLX, average [AVG] power EXT, AVG power FLX).

**Table 5:** Isokinetic Muscle Endurance Indices for the CoQ10 and Placebo (CON) groups.

**Variable**	**Time**	**CoQ10**	**Placebo**	**Significance**	
Peak Torque EXT (N/m)	Baseline	132 ± 42	110 ± 38	Group	0.181
	Day 0	127 ± 37	110 ± 34	Time	0.265
	Day 14	132 ± 34	107 ± 35	G × T	0.495
					
Peak Torque FLX (N/m)	Baseline	72 ± 28	65 ± 26	Group	0.218
	Day 0	75 ± 28	66 ± 28	Time	0.534
	Day 14	73 ± 24	65 ± 28	G × T	0.488
					
Total Work EXT (N/m)	Baseline	4047 ± 1218	3391 ± 992	Group	0.826
	Day 0	3955 ± 1114	3352 ± 936	Time	0.308
	Day 14	4073 ± 1116	3242 ± 921	G × T	0.442
					
Total Work FLX (N/m)	Baseline	2213 ± 847	1962 ± 891	Group	0.127
	Day 0	2203 ± 877	1996 ± 963	Time	0.493
	Day 14	2230 ± 822	1866 ± 839	G × T	0.591
					
Work Fatigue EXT (%)	Baseline	48 ± 15	41 ± 17	Group	0.112
	Day 0	46 ± 14	37 ± 19	Time	0.160
	Day 14	48 ± 11	36 ± 20	G × T	0.309
					
Work Fatigue FLX (%)	Baseline	46 ± 20	43 ± 29	Group	0.132
	Day 0	51 ± 14	35 ± 30	Time	0.534
	Day 14	51 ± 16	44 ± 14	G × T	0.192
					
AVG Power EXT (Watts)	Baseline	117 ± 36	95 ± 30	Group	0.476
	Day 0	115 ± 34	95 ± 27	Time	0.315
	Day 14	117 ± 33	95 ± 30	G × T	0.351
					
AVG Power FLX (Watts)	Baseline	61 ± 25	52 ± 25	Group	0.106
	Day 0	62 ± 26	54 ± 27	Time	0.334
	Day 14	62 ± 24	52 ± 26	G × T	0.402

Data are means and ± standard deviations of mean

### Anaerobic capacity assessment

A 30 second Wingate anaerobic capacity test was performed at baseline, day 0, and day 14 to assess indices of anaerobic performance (see Table 6). No statistically significant interactions were observed among groups in anaerobic capacity indices (peak power, mean power, rate to fatigue, or total work). A trend towards a significant main effect for time was observed in rate to fatigue performance (p = 0.06), indicating an increase in rate to fatigue from baseline (T1) to day 14 (T3).

**Table 6:** Wingate Anaerobic Power Indices for the CoQ10 and Placebo (CON) groups.

**Variable**	**Time**	**CoQ10**	**Placebo**	**Significance**	
Peak Power (Watts)	Baseline	1162 ± 436	1013 ± 417	Group	0.359
	Day 0	1169 ± 421	1079 ± 416	Time	0.138
	Day 14	1209 ± 420	1069 ± 400	G × T	0.587
					
Mean Power (Watts)	Baseline	592 ± 210	535 ± 181	Group	0.427
	Day 0	594 ± 209	545 ± 180	Time	0.137
	Day 14	603 ± 211	548 ± 181	G × T	0.883
					
Rate to Fatigue (Watts)	Baseline	33 ± 14	27 ± 15	Group	0.239
	Day 0	34 ± 15	30 ± 15	Time	0.060
	Day 14	35 ± 15	29 ± 14	G × T	0.623

Data are means and ± standard deviations of mean

### Aerobic capacity assessment

A maximal graded exercise test was performed at baseline, day 0, and day 14 to assess aerobic capacity and performance (see Table 7). A trend towards a significant group × time interaction was observed in time to exhaustion (p = 0.06), indicating the CoQ10 group exercised longer compared to the CON group following 2 weeks of supplementation. No statistically significant interactions were observed across groups in other indices of aerobic capacity (i.e., oxygen consumption, RER, or ventilatory anaerobic threshold).

**Table 7:** Aerobic Power Indices for the CoQ10 and Placebo (CON) groups.

**Variable**	**Time**	**CoQ10**	**Placebo**	**Significance**	
Oxygen Consumption (mL·kg^-1^·min^-1^)	Baseline	42.3 ± 9.1	43.9 ± 9.0	Group	0.358
	Day 0	42.9 ± 8.5	45.5 ± 7.9	Time	0.815
	Day 14	42.1 ± 8.5	43.4 ± 9.6	G × T	0.293
					
Maximal Respiratory Exchange Ratio	Baseline	1.16 ± 0.09	1.17 ± 0.08	Group	0.564
	Day 0	1.18 ± 0.09	1.19 ± 0.04	Time	0.134
	Day 14	1.16 ± 0.09	1.16 ± 0.11	G × T	0.546
					
Time to exhaustion (Min)	Baseline	12.9 ± 2.4	13.6 ± 2.3	Group	0.332
	Day 0	13.1 ± 2.8	13.8 ± 2.1	Time	0.110
	Day 14	13.3 ± 2.3	13.7 ± 2.4	G × T	0.060
					
Ventilatory Threshold (% VO_2_max)	Baseline	70.4 ± 12.9	71.35 ± 11.6	Group	0.210
	Day 0	70.7 ± 11.4	72.30 ± 11.4	Time	0.951
	Day 14	72.1 ± 12.0	70.00 ± 11.9	G × T	0.536

Data are means and ± standard deviations of mean

### Markers of oxidative stress

Table 8 presents markers of oxidative stressed evaluated in this study. A significant main effect for time was observed in serum SOD levels, indicating an increase following all exercise performance measurements on day 0 (T2) and day 14 (T3). However, a trend towards a significant group × time interaction (p = 0.06) was revealed with subsequent post-hoc analysis indicating that although SOD levels increased over time. SOD levels after the isokinetic muscle endurance test were significantly lower in the CoQ10 compared to CON group (p < 0.05). A significant main effect for time was also observed in serum 8-ISO and MDA levels (p < 0.05 and p < 0.001, respectively) indicating an increase over time following all exercise performance measurements on day 0 (T2) and day 14 (T3). Similar to SOD levels, a significant group × time interaction (p < 0.05) was also revealed on day 0. However, post-hoc analysis indicated significantly lower MDA levels in the CON group compared to CoQ10 group following the anaerobic Wingate test and aerobic capacity test (p < 0.05). No other significant interactions were observed.

**Table 8:** Time Course of Markers of Oxidative Stress for the CoQ10 and Placebo (CON) subjects taken immediately after each performance test.

**Variable**	**Time**	**CoQ10**	**Placebo**	**Significance**	
Superoxide Dismutase (SOD) (U/ml)	Baseline T2	1.52 ± 0.31	1.59 ± 0.27	Group	0.103
	Isokinetic T2	1.56 ± 0.24	1.80 ± 0.39	Time	0.001
	Wingate T2	1.75 ± 0.23	1.70 ± 0.22	G × T	0.060
	GXT T2	1.68 ± 0.17	1.70 ± 0.26		
					
	Baseline T3	1.61 ± 0.40	1.77 ± 0.52	Group	0.296
	Isokinetic T3	1.74 ± 0.37	1.90 ± 0.54	Time	0.001†
	Wingate T3	1.91 ± 0.39	2.06 ± 0.51	G × T	0.966
	GXT T3	1.81 ± 0.42	1.98 ± 0.50		
					
Thiobarbituric Acid Reactive Substance (TBARS) (μM)	Baseline T2	2.79 ± 1.02	2.50 ± 1.34	Group	0.042
	Isokinetic T2	4.11 ± 1.71	3.24 ± 1.69	Time	0.001
	Wingate T2	4.72 ± 2.14	3.35 ± 1.57†	G × T	0.018†
	GXT T2	5.09 ± 2.23	3.62 ± 1.90†		
					
	Baseline T3	3.03 ± 1.03	3.12 ± 1.26	Group	0.540
	Isokinetic T3	4.28 ± 1.72	4.23 ± 1.47	Time	0.001
	Wingate T3	4.25 ± 1.17	4.85 ± 2.11	G × T	0.534
	GXT T3	5.32 ± 2.09	5.52 ± 1.89		
					
8-Isoprostane (pg/ml)	Baseline T2	76 ± 28	78 ± 33	Group	0.616
	Isokinetic T2	86 ± 63	88 ± 61	Time	0.050
	Wingate T2	105 ± 72	99 ± 63	G × T	0.431
	GXT T2	108 ± 67	110 ± 62		
					
	Baseline T3	84 ± 46	79 ± 35	Group	0.334
	Isokinetic T3	101 ± 37	124 ± 75	Time	0.001
	Wingate T3	114 ± 53	122 ± 69	G × T	0.553
	GXT T3	99 ± 49	111 ± 87		

Data are means and ± standard deviations of mean.

† represents p < 0.05 difference between groups

### Plasma and Muscle CoQ10 Concentration

**Figure 2:**
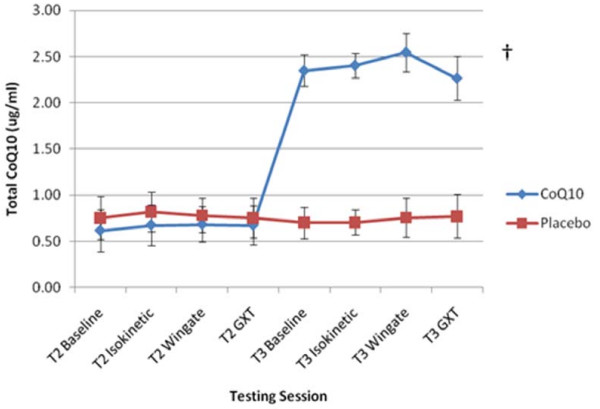
**Time course of plasma CoQ10 concentration**. Data (mean ± SD) represents total plasma CoQ10 concentration (ug/ml) for both CoQ10 and placebo control groups taken prior to, and immediately after each performance test on day 0 (T2) and day 14 (T3). † represents (p < 0.05) difference from control.

Total plasma CoQ10 content data are presented in Figure 2. A significant main effect for time was observed in plasma CoQ10 (p < 0.001). A significant group × time interaction was also observed in plasma CoQ10 (p < 0.001) indicating a significant increase in plasma CoQ10 in the CoQ10-supplemented group compared to CON. No other significant effects were observed.

**Figure 3:**
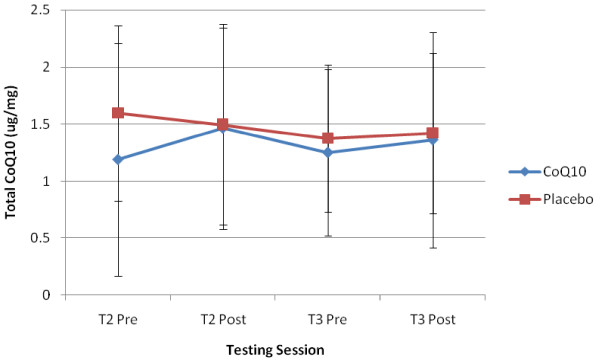
**Time course of muscle CoQ10 concentration**. Data (mean ± SD) represents total muscle CoQ10 concentration (ug/mg muscle) for both CoQ10 and placebo control groups taken pre and immediately post the final performance test on day 0 (T2) and day 14 (T3).

Muscle total CoQ10 content data are presented in Figure 3. A trend towards a significant group × time interaction (p = 0.098) was observed on day 0 (T2), indicating higher muscle CoQ10 levels (mcg/mg) following an acute ingestion of CoQ10. A trend towards a significant group × gender (p = 0.09) and training status × gender (p = 0.06) interaction were observed, indicating higher levels of CoQ10 in both trained and male subjects respectively. No significant interactions were observed following chronic supplementation.

### Correlation analysis

Following acute ingestion of CoQ10, plasma CoQ10 was significantly correlated to muscle CoQ10 levels; maximal oxygen consumption; and treadmill time to exhaustion. No other significant correlations were observed following acute and chronic supplementation (see Table 9).

**Table 9:** Correlation Data with Baseline Plasma CoQ10 levels

**Variable**	**Significance**	
Muscle Total CoQ10 (mcg/mg) T2 pre ¥	r value	0.423
	p value	0.007
		
Muscle Total CoQ10 (mcg/mg) T2 post	r value	0.189
	p value	0.250
		
Muscle Total CoQ10 (mcg/mg) T3 pre	r value	0.247
	p value	0.125
		
Muscle Total CoQ10 (mcg/mg) T3 post	r value	0.244
	p value	0.134
		
Oxygen Consumption (mL·kg^-1^·min^-1^) T1 ¥	r value	0.389
	p value	0.013
		
Oxygen Consumption (mL·kg^-1^·min^-1^) T2 ¥	r value	0.381
	p value	0.017
		
Oxygen Consumption (mL·kg^-1^·min^-1^) T3 ¥	r value	0.429
	p value	0.006
		
Time to exhaustion (Min) T1 ¥	r value	0.419
	p value	0.007
		
Time to exhaustion (Min) T2 ¥	r value	0.454
	p value	0.004
		
Time to exhaustion (Min) T3 ¥	r value	0.485
	p value	0.001

¥ represents significantly correlated to baseline plasma CoQ10 levels

## Discussion

The results from the present study demonstrate that a fast-melt form of CoQ10 is a safe and effective supplement that prolongs exercise performance in healthy individuals. Further, acute CoQ10 supplementation increased total CoQ10 concentration within the skeletal muscle and lowered plasma oxidative stress (SOD) during and following exercise. Though there is evidence in the literature to suggest a possible therapeutic role for CoQ10 in human diseases such as CHF, the potential ergogenic value in healthy trained and untrained individuals is less clear. Studies that have reported improvements in aerobic power, anaerobic threshold, and/or recovery following CoQ10 supplementation have not generally been published in peer-reviewed journals [4,13]. To our knowledge, this is the first study to demonstrate both improvements in exercise performance and increased muscle CoQ10 concentration in healthy trained and untrained humans following CoQ10 supplementation.

In the current study, both acute and chronic CoQ10 supplementation had no significant effect of indices of muscle endurance and anaerobic capacity as determined by an isokinetic 50-repetition test and a 30-second Wingate anaerobic capacity test. Similarly, no significant differences were observed among groups in maximal oxygen uptake (VO_2max_) or ventilatory anaerobic threshold following CoQ10 supplementation. These findings are in line with a number of previous studies [3,15,16,19] which have also failed to demonstrate performance-enhancing effects in trained athletes and/or untrained individuals following CoQ10 supplementation. Recently, Zhou and colleagues [3] investigated the effects of 4 weeks of CoQ10 supplementation on aerobic power, ventilatory threshold and exercise economy of healthy males. Results showed no significant changes in aerobic performance (VO_2max_, ventilatory threshold) or exercise economy in response to supplementation. Further, though CoQ10 concentration was elevated within the plasma, no significant increases were observed in the muscle. Thus, the authors concluded that the lack of ergogenic benefit was likely due to an inability of supplementation protocol to increase CoQ10 concentration within the muscle [3].

**Figure 4:**
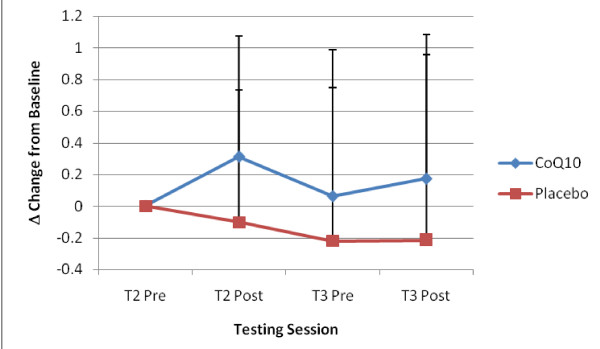
**Delta changes from baseline for muscle CoQ10 concentration**. Data (mean ± SD) represents delta scores (post minus pre values) for each performance test (Isokinetic, Wingate, Cardiopulmonary), both CoQ10 and placebo control groups on day 0 (T2) and day 14 (T3).

Similar to Zhou and coworkers [3], results from the present study showed a significant increase in plasma CoQ10 concentration following chronic supplementation. However, contrary to the observations of Zhou et al., [3], the present study showed a strong trend (p = 0.06) for increased time to exhaustion following CoQ10 supplementation. In addition, acute CoQ10 supplementation tended to increase CoQ10 levels within the muscle, albeit not significantly (p = 0.09). Though no significant changes were observed following chronic supplementation, it is evident from Figure 4 that muscle CoQ10 concentration was generally higher after baseline in the CoQ10-supplemented group compared to placebo group. In fact, muscle CoQ10 levels declined in the placebo group over the 2-week period. These findings indicate that ingestion of a fast-melt form of CoQ10 will increase plasma availability of CoQ10 and may also influence muscle concentrations on an acute and/or chronic basis. However, more research is needed before definitive conclusions can be drawn.

In the present study, the pre-supplementation concentration of muscle CoQ10 (14.96 ± 10.3 μg/g tissue) was comparable to those reported by Boitier et al., [32] (19.0 ± 4.8 μg/g) and Pastore et al., [33] (18.34 ± 5.5 pmol/mg tissue). Conversely, Lamperti and colleagues [34] observed higher CoQ10 mean values of 27.64 ± 4.4 μg/g fresh tissue, following analysis of 121 healthy human muscle samples. In the current study, muscle tissue was initially frozen and then subsequently analyzed 2–3 months following the final data collection. This could explain the lower mean values observed in the present study compared to those reported by Lamperti et al., [34]. Approximately one half of CoQ10 in the body resides in the inner mitochondrial membrane [10]. CoQ10 is normally localized in the central hydrophobic portion of the membrane and thus, it has been suggested that its concentration may not be able to increase freely within the membrane due to physical limits and potential destabilization of the bilayer structure [35]. A number of human studies support this view that uptake of exogenous CoQ10 (e.g., 120–150 mg of CoQ10 for 4–6 weeks) by tissues other than plasma and liver is very low or absent [3,16,36]. However, recent findings in young rodents argue against such a viewpoint, and suggest dietary supplementation with CoQ10 can lead to elevated CoQ10 levels within the mitochondria of the skeletal muscle [37]. One possible factor that may limit augmentation of CoQ10 in tissues in response to exogenous intake (oral intake) is duration.

Although the dosage in the current study was similar to previous studies (i.e., 200 mg vs 150 mg), most studies only examined the chronic effects of CoQ10 supplementation on intramuscular CoQ10 levels [3,16,36]. In the current study, muscle CoQ10 concentration increased within about 2 hours of ingestion. Muscle CoQ10 concentrations remained above baseline values after 2 weeks of supplementation albeit to a much lesser degree (Figure 4). It could be suggested that pharmacokinetics properties of CoQ10 uptake into the muscle is similar to that of creatine monohydrate. In this regard, following chronic creatine supplementation, there appears to be a maximal limit and/or down regulation of creatine transporters, eventually leading to a plateau or even a decrease in intramuscular creatine levels over time [38]. Such an effect could explain why previous studies have failed to show any increases in muscle CoQ10 concentration following 4–6 weeks of supplementation. However, whether a maximal limit and/or down regulation in the transport of CoQ10 into the muscle occurs following chronic CoQ10 supplementation is highly speculative and requires further investigation

It is well established that CoQ10 is synthesized de novo in all tissues, and hence, under normal circumstances, most tissues are not dependent on an exogenous supply of CoQ10. However, under certain conditions such as oxidative stress and aging, endogenous production may not meet the demands for CoQ10 [39], and thus uptake may be increased. In the current study, it is evident that intramuscular CoQ10 levels increased following each testing session (T2 Pre to Post and T3 Pre to Post); while the placebo group showed an overall decrease in intramuscular CoQ10 levels (Figure 4). This may suggest an enhanced uptake of exogenous CoQ10 from the plasma as a consequence of increased metabolism in active tissue. However, this will require further investigation. An alternative hypothesis is that since the new "fast-melt" CoQ10 formulation has demonstrated enhanced absorption kinetics into the blood stream compared to previous commercially available formulations [21], the increased bioavailability may enhance greater uptake into the muscle. However, additional research will be needed to examine this hypothesis as well.

Increased muscle CoQ10 concentration could explain the increased time to exhaustion observed in the current study. It is has been previously suggested that the CoQ10 redox shuttle is a rate limiting step in the oxidative phosphorylation pathway [40]. Hence, an increase in CoQ10 content within the mitochondria would potentially enhance the oxidative phosphorylation process and subsequently prolong exercise performance [3]. It is well known that approximately 3 minutes is required for oxygen consumption to reach a steady state at the beginning of a moderate exercise at constant workload [41]. One factor that determines how quickly oxygen consumption rises is mitochondrial density (i.e. the amount of enzymes in oxidative phosphorylation) [42]. Zhou and colleagues [3] demonstrated a trend for decreased oxygen deficit (an indirect indicator of mitochondrial function) in response to 4 weeks CoQ10 supplementation. However, due to a small sample size and no change in muscle CoQ10 concentration, authors were unable to determine any significance behind such an observation. In the current study, although oxygen deficit was not measured, time to exhaustion and muscle CoQ10 concentration was increased following supplementation. The increased time to exhaustion observed in the present study may be indirect evidence of enhanced oxidative phosphorylation. However, further research examining mitochondrial enzymes would be needed confirm such observations.

In addition, it is speculated that increased muscle performance might be due to the antioxidant effect of CoQ10 supplementation and/or its potential action on the central nervous system [13]. In a single-blind fashion, Bonetti and colleagues, [13] reported an increase in maximal workload completed at exhaustion following 8 weeks of CoQ10 supplementation [100 mg/day]. No significant changes in VO_2max _and anaerobic threshold levels were observed. The authors concluded that the increased maximum workload at exhaustion, without signs of improved aerobic power (VO2max) and/or ventilator threshold (similar to that observed in the current study), could be related to the protective activity of CoQ10 on the muscular membrane (antioxidant effect) [13]. In the current study, only serum markers of oxidative stress (i.e., SOD, 8-Isoprostane, and MDA activity) were measured. SOD activity is a marker of cellular antioxidant defenses and an indirect marker of oxidative damage (i.e. an increase in serum SOD activity indicates an increased presence of superoxide radical in the serum, hence, increased oxidative stress). 8-Isoprostane is a member of the eicosanoid family and is produced by random oxidation of tissue phospholipids by oxygen radicals and is therefore a direct marker of oxidative stress. MDA is a naturally occurring product of lipid peroxidation and similar to 8-Isoprostane, is a direct maker of oxidative stress.

The baseline values for each of these markers were similar to previous studies [43,44]. As expected, all markers of oxidative stress increased over time during each testing session (T2 and T3). Acute and chronic CoQ10 supplementation had no significant effect on 8-isoprostane levels during exercise. However, significant differences were observed in serum SOD and MDA among groups. In this regard, serum SOD levels were significantly lower in the CoQ10 group compared to placebo group (p < 0.05) following the isokinetic muscle endurance test. Additionally, serum MDA levels were significantly higher in the CoQ10 compared to placebo group (p < 0.05) following the Wingate anaerobic capacity test and aerobic capacity test. Although previous research has demonstrated decreased oxidative stress following CoQ10 supplementation [45], such effects have only been observed in diseased states. Indeed, a recent study showed improved extracellular SOD activity and endothelium-dependent vasodilation following oral ingestion of CoQ10 (300 mg/day for 4 weeks) in patients with coronary artery disease [46]. Therefore, this is the first study to the author's knowledge, to show a decrease in SOD activity following CoQ10 supplementation in healthy individuals.

It is not apparent why serum MDA levels were significantly higher in the CoQ10 group compared to placebo group following the Wingate anaerobic capacity test and aerobic capacity test. It has been suggested that although TBARS is most widely employed assay used to determine lipid peroxidation, that compounds other than MDA may interfere with the specificity of this assay [47]. Therefore, any changes in serum MDA concentration could be due to interference from other compounds rather than lipid peroxidation. However, whether this explains the different responses observed in markers of oxidative stress requires further investigation.

In conclusion, the present study demonstrated that a fast-melt CoQ10 formulation appears to be a safe dietary supplement that tended to increase the duration of exercise to exhaustion in healthy untrained and trained individuals. Acute supplementation with CoQ10 resulted in higher muscle CoQ10 concentration and lower serum SOD oxidative stress during and following exercise. The observed increase in exercise performance could be due to a combination of an enhanced oxidative phosphorylation within the mitochondria and/or enhanced antioxidant protection. However, further research is clearly needed to elucidate such mechanisms.

## Competing interests

This study was funded by Pharma Base, S.A. (Switzerland) through an unrestricted research grant to Baylor University. All researchers involved independently collected, analyzed, and interpreted the results from this study and have no financial interests concerning the outcome of this investigation. The results from this study do not constitute endorsement by the authors and/or their institutions concerning nutrients investigated.

## Authors' contributions

Matthew Cooke assisted in designing the study, writing the grant, study coordination, data analysis, and manuscript preparation. Mike Iosia assisted in data collection and supervision of the study. Thomas Buford assisted in data collection. Brian Shelmadine assisted in data collection. Geoffrey Hudson assisted in data collection. Chad Kerksick assisted in data collection. Chris Rasmussen is director of the exercise and sport nutrition lab. Mike Greenwood assisted in research design and consultation. Brian Leutholtz assisted in research design and consultation. Darryn Willoughby assisted in research design and consultation. Richard B. Kreider assisted in the design of the study, data analysis, manuscript preparation, obtained the grant, and served as PI of study. All authors read and approved the final manuscript.

## References

[B1] Linnane AW, Kopsidas G, Zhang C, Yarovaya N, Kovalenko S, Papakostopoulos P, Eastwood H, Graves S, Richardson M (2002). Cellular redox activity of coenzyme Q10: effect of CoQ10 supplementation on human skeletal muscle. Free Radic Res.

[B2] Butler MG, Dasouki M, Bittel D, Hunter S, Naini A, DiMauro S (2003). Coenzyme Q10 levels in Prader-Willi syndrome: comparison with obese and non-obese subjects. Am J Med Genet A.

[B3] Zhou S, Zhang Y, Davie A, Marshall-Gradisnik S, Hu H, Wang J, Brushett D (2005). Muscle and plasma coenzyme Q10 concentration, aerobic power and exercise economy of healthy men in response to four weeks of supplementation. J Sports Med Phys Fitness.

[B4] Rosenfeldt F, Hilton D, Pepe S, Krum H (2003). Systematic review of effect of coenzyme Q10 in physical exercise, hypertension and heart failure. Biofactors.

[B5] Crane FL (2001). Biochemical functions of coenzyme Q10. J Am Coll Nutr.

[B6] Niki E (1997). Mechanisms and dynamics of antioxidant action of ubiquinol. Mol Aspects Med.

[B7] Jones K, Hughes K, Mischley L, McKenna DJ (2002). Coenzyme Q-10: efficacy, safety, and use. Altern Ther Health Med.

[B8] Keith M, Geranmayegan A, Sole MJ, Kurian R, Robinson A, Omran AS, Jeejeebhoy KN (1998). Increased oxidative stress in patients with congestive heart failure. J Am Coll Cardiol.

[B9] Shults CW, Oakes D, Kieburtz K, Beal MF, Haas R, Plumb S, Juncos JL, Nutt J, Shoulson I, Carter J (2002). Effects of coenzyme Q10 in early Parkinson disease: evidence of slowing of the functional decline. Arch Neurol.

[B10] Greenberg S, Frishman WH (1990). Co-enzyme Q10: a new drug for cardiovascular disease. J Clin Pharmacol.

[B11] Tran MT, Mitchell TM, Kennedy DT, Giles JT (2001). Role of coenzyme Q10 in chronic heart failure, angina, and hypertension. Pharmacotherapy.

[B12] Zuliani U, Bonetti A, Campana M, Cerioli G, Solito F, Novarini A (1989). The influence of ubiquinone (Co Q10) on the metabolic response to work. J Sports Med Phys Fitness.

[B13] Bonetti A, Solito F, Carmosino G, Bargossi AM, Fiorella PL (2000). Effect of ubidecarenone oral treatment on aerobic power in middle-aged trained subjects. J Sports Med Phys Fitness.

[B14] Littarru GP (1993). Biomedical and clinical aspects of coenzyme Q. Clin Investig.

[B15] Braun B, Clarkson PM, Freedson PS, Kohl RL (1991). Effects of coenzyme Q10 supplementation on exercise performance, VO2max, and lipid peroxidation in trained cyclists. Int J Sport Nutr.

[B16] Laaksonen R, Fogelholm M, Himberg JJ, Laakso J, Salorinne Y (1995). Ubiquinone supplementation and exercise capacity in trained young and older men. Eur J Appl Physiol Occup Physiol.

[B17] Porter DA, Costill DL, Zachwieja JJ, Krzeminski K, Fink WJ, Wagner E, Folkers K (1995). The effect of oral coenzyme Q10 on the exercise tolerance of middle-aged, untrained men. Int J Sports Med.

[B18] Malm C, Svensson M, Ekblom B, Sjodin B (1997). Effects of ubiquinone-10 supplementation and high intensity training on physical performance in humans. Acta Physiol Scand.

[B19] Weston SB, Zhou S, Weatherby RP, Robson SJ (1997). Does exogenous coenzyme Q10 affect aerobic capacity in endurance athletes?. Int J Sport Nutr.

[B20] Kaikkonen J, Tuomainen TP, Nyyssonen K, Salonen JT (2002). Coenzyme Q10: absorption, antioxidative properties, determinants, and plasma levels. Free Radic Res.

[B21] Joshi SS, Sawant SV, Shedge A, Halpner AD (2003). Comparative bioavailability of two novel coenzyme Q10 preparations in humans. Int J Clin Pharmacol Ther.

[B22] Kawabata Y, Senda M, Oka T, Yagata Y, Takahara Y, Nagashima H, Inoue H (2000). Measurement of fatigue in knee flexor and extensor muscles. Acta Med Okayama.

[B23] Pincivero DM, Gear WS, Sterner RL (2001). Assessment of the reliability of high-intensity quadriceps femoris muscle fatigue. Med Sci Sports Exerc.

[B24] Fielding RA, Frontera WR, Hughes VA, Fisher EC, Evans WJ (1997). The reproducibility of the Bruce protocol exercise test for the determination of aerobic capacity in older women. Med Sci Sports Exerc.

[B25] ACSM (2000). ACSM's Guidelines for Exercise Testing and Prescription.

[B26] Klesges RC, Ward KD, Shelton ML, Applegate WB, Cantler ED, Palmieri GM, Harmon K, Davis J (1996). Changes in bone mineral content in male athletes. Mechanisms of action and intervention effects. JAMA.

[B27] (1992). Diagnostic and therapeutic technology assessment. Measurement of bone density with dual-energy X-ray absorptiometry (DEXA). JAMA.

[B28] Campbell B, Roberts M, Kerksick C, Wilborn C, Marcello B, Taylor L, Nassar E, Leutholtz B, Bowden R, Rasmussen C (2006). Pharmacokinetics, safety, and effects on exercise performance of l-arginine alpha-ketoglutarate in trained adult men. Nutrition.

[B29] Tang PH, Miles MV, DeGrauw A, Hershey A, Pesce A (2001). HPLC analysis of reduced and oxidized coenzyme Q(10) in human plasma. Clin Chem.

[B30] Rousseau G, Varin F (1998). Determination of ubiquinone-9 and 10 levels in rat tissues and blood by high-performance liquid chromatography with ultraviolet detection. J Chromatogr Sci.

[B31] Pagana KD, Pagana TJ (2006). Mosby's manual of diagnostic and laboratory tests.

[B32] Boitier E, Degoul F, Desguerre I, Charpentier C, Francois D, Ponsot G, Diry M, Rustin P, Marsac C (1998). A case of mitochondrial encephalomyopathy associated with a muscle coenzyme Q10 deficiency. J Neurol Sci.

[B33] Pastore A, Giovamberardino GD, Bertini E, Tozzi G, Gaeta LM, Federici G, Piemonte F (2005). Simultaneous determination of ubiquinol and ubiquinone in skeletal muscle of pediatric patients. Anal Biochem.

[B34] Lamperti C, Naini A, Hirano M, De Vivo DC, Bertini E, Servidei S, Valeriani M, Lynch D, Banwell B, Berg M (2003). Cerebellar ataxia and coenzyme Q10 deficiency. Neurology.

[B35] Turunen M, Swiezewska E, Chojnacki T, Sindelar P, Dallner G (2002). Regulatory aspects of coenzyme Q metabolism. Free Radic Res.

[B36] Svensson M, Malm C, Tonkonogi M, Ekblom B, Sjodin B, Sahlin K (1999). Effect of Q10 supplementation on tissue Q10 levels and adenine nucleotide catabolism during high-intensity exercise. Int J Sport Nutr.

[B37] Kamzalov S, Sumien N, Forster MJ, Sohal RS (2003). Coenzyme Q intake elevates the mitochondrial and tissue levels of Coenzyme Q and alpha-tocopherol in young mice. J Nutr.

[B38] Guerrero-Ontiveros ML, Wallimann T (1998). Creatine supplementation in health and disease. Effects of chronic creatine ingestion in vivo: down-regulation of the expression of creatine transporter isoforms in skeletal muscle. Mol Cell Biochem.

[B39] Bhagavan HN, Chopra RK (2006). Coenzyme Q10: absorption, tissue uptake, metabolism and pharmacokinetics. Free Radic Res.

[B40] Hackenbrock CR, Chazotte B, Gupte SS (1986). The random collision model and a critical assessment of diffusion and collision in mitochondrial electron transport. J Bioenerg Biomembr.

[B41] Xu F, Rhodes EC (1999). Oxygen uptake kinetics during exercise. Sports Med.

[B42] Barstow TJ (1994). Characterization of VO2 kinetics during heavy exercise. Med Sci Sports Exerc.

[B43] Waring WS, Convery A, Mishra V, Shenkin A, Webb DJ, Maxwell SR (2003). Uric acid reduces exercise-induced oxidative stress in healthy adults. Clin Sci (Lond).

[B44] Tauler P, Sureda A, Cases N, Aguilo A, Rodriguez-Marroyo JA, Villa G, Tur JA, Pons A (2006). Increased lymphocyte antioxidant defences in response to exhaustive exercise do not prevent oxidative damage. J Nutr Biochem.

[B45] Littarru GP, Tiano L (2007). Bioenergetic and antioxidant properties of coenzyme Q10: recent developments. Mol Biotechnol.

[B46] Tiano L, Belardinelli R, Carnevali P, Principi F, Seddaiu G, Littarru GP (2007). Effect of coenzyme Q10 administration on endothelial function and extracellular superoxide dismutase in patients with ischaemic heart disease: a double-blind, randomized controlled study. Eur Heart J.

[B47] Lykkesfeldt J (2007). Malondialdehyde as biomarker of oxidative damage to lipids caused by smoking. Clin Chim Acta.

